# Understanding acute kidney injury in low resource settings: a step forward

**DOI:** 10.1186/1471-2369-16-5

**Published:** 2015-01-16

**Authors:** Shuchi Anand, Dinna N Cruz, Fredric O Finkelstein

**Affiliations:** Stanford University, Palto Alto, CA USA; Division of Nephrology-Hypertension, Department of Medicine, University of California, San Diego, USA; Yale University, 136 Sherman Avenue, New Haven, USA

## Abstract

Attention has recently been focused on addressing the problem of acute kidney injury in both the developed and developing world. Little information is actually available on the incidence and management of AKI in low resource settings. Thus, the paper by Bagasha in the current issue of *BMC Nephrology* makes an important contribution to our understanding of this serious and potentially remediable problem.

## Background

The International Society of Nephrology has recently set a goal of eliminating preventable or treatable deaths from acute kidney injury (AKI) by 2025—the “0X25” initiative. Programmatic implementation in low resource settings (LRS) is a key mission of this initiative. But a major challenge in designing effective programs to treat AKI in LRS is that we do not know the extent and nature of the problem.

### Main text

Few studies describe the epidemiology of AKI in LRS [1–3]. In a 2013 meta-analysis examining 154 studies on the incidence of AKI, the authors could identify only two adult studies from LRS which used a standard definition of AKI with sample sizes exceeding 500 adults or 50 children [1]. A number of factors contribute to this paucity of good quality data. In LRS, late presentation of patients to tertiary care centers is common. Often, limited resources force a difficult choice between spending money on serial laboratory tests or treatment, resulting in ascertainment bias. Even when an attempt is made to study AKI systematically, the limitations on laboratory services with rapid turn-around sometimes lead to use of alternate AKI definitions, since current consensus AKI definitions are partly based on serial creatinine measurements. Furthermore, most centers still use paper rather than electronic medical records, making data extraction less efficient and multicenter collaboration more difficult. As a result, many studies are often single center with small sample sizes [2]. They often do not make it to publication in high profile journals, but instead are published in regional journals which are not readily accessible to the general medical community.

Thus, the study published in this issue of *BMC Nephrology* by Bagasha et al. begins to fill an important data gap [4]. Using the Acute Kidney Injury Network definition, the study describes the epidemiology and correlates of AKI among 387 adult patients admitted to Mulago National Referral Hospital (the largest hospital in Uganda) who fit criteria for a diagnosis of sepsis. The prevalence of AKI as assessed at a single time point was 16%, with 46% of patients developing severe AKI. Overall mortality was 21%.

The numbers presented in this study, while stark, are likely underestimates for several reasons. The authors excluded patients with chronic kidney disease, a group at high-risk for AKI in the setting of sepsis. The assessment for AKI occurred only at enrollment, and patients who may have developed AKI in their clinical course were not included. Moving away from a tertiary care center, we can speculate that in rural or other urban centers without academic support, the diagnosis of AKI is often missed or delayed, and outcomes will likely be even worse.

Nonetheless, the study by Bagasha et al. provides an important snapshot of the patients admitted for sepsis likely to develop AKI in Uganda: most are young, have HIV, and may well have used herbal medications prior to admission. We can extrapolate that such a patient will likely *not* receive intensive unit care, and if he/she develops severe AKI, the likelihood of death will approach 40%. Dialysis initiation will occur rarely or not at all, even at the largest referral center in Uganda.

This picture contrasts with the one seen in high-resource settings, where AKI is most often hospital acquired, in patients with multiorgan failure and/or multiple co-morbidities who have been exposed to polypharmacy and invasive procedures. In a study of 22 centers in the U.S., Canada, and Saudi Arabia, patients with AKI were often critically-ill, with close to 40% having 2 or more co-morbidities [5]. Average age of patients with sepsis-related AKI exceeded 60 years in this and other reports from Italy [6], Germany [7], and New Zealand [8]. Dialytic support was used in 2-10% of AKI patients [1, 6–8].

The differences between AKI in LRS, as noted in the Bagasha et al. study and other single center studies, and high resource settings are important to note for several reasons. First, AKI is potentially more “preventable and treatable”. Appropriate hygiene may decrease the incidence and spread of diarrheal diseases. Volume depletion, when recognized sufficiently early, could potentially be addressed by inexpensive oral rehydration strategies in the community or intravenous fluids in the hospital. Given that the majority of patients in the study by Bagasha et al. had received <1L of fluid at the time of diagnosis, how many episodes of AKI could have been prevented if appropriate hemodynamic support were provided? Early administration of antibiotics or antimalarials could also help address potentially remediable diseases. Avoidance of nephrotoxins, another cornerstone of the management of critically ill patients, can also make a major difference in limiting the incidence and/or progression of AKI, especially given that close to 25% of patients with AKI used herbal medications prior to their admission to the hospital.

Second, the patients are young. Nearly half of patients experiencing AKI in the Bagasha et al. study were younger than 40 years; a similarly young age distribution has been reported from South Africa [9] and Sri Lanka [10]. Few patients have co-morbidities other than HIV. Often the kidney is the only failed organ. These factors contribute to a higher likelihood of full renal and overall recovery, and return of young individuals to become active members of society.

Third, if patients do progress to AKI in LRS, many have no options for dialysis therapy. Dialytic support is limited by the lack of resources, lack of available equipment, and lack of funding necessary to support such therapy. Patients are often asked to pay the high costs of treatment—costs which are generally beyond their means. These sobering facts make the prevention or at least the mitigation of AKI even more important.

Thus, the 0X25 initiative has been embraced by the nephrology community worldwide, and strategies are actively being organized to address the problem of AKI in LRS. The cornerstones of the program involve identifying the causes of AKI, understanding what resources are available to treat patients at risk for AKI, developing education programs to increase the awareness of the importance of early diagnosis and treatment of AKI, and then developing appropriate treatment strategies.

Understanding how to approach education and treatment strategies requires that plans be thought of in the structural, cultural, and financial context of the setting in which these programs are being developed. Thus, in a thoughtful and detailed review of the Millenium Village Project, Nina Munk provides a cautionary story [11]. Munk, who spent 6 years examining the impact of the Millenium Project in Africa, describes the dangers inherent in imposing outside theories on the complex and ever-changing lives of African villagers. She reflects on the cultural difficulties that outside aid efforts are likely to encounter in rural Africa. In terms of renal replacement therapy, the use of peritoneal dialysis (PD) is now being again recognized as an acceptable, cost-efficient, and technologically simple therapy to be used not only in LRS but in high income countries as well [12, 13]. Thus, Chionh et al. have suggested that outcomes with PD for the treatment of AKI are as good as with extracorporeal therapies [13]. Comprehensive guidelines for the use of PD to treat patients (children and adults) with AKI have recently been published [13]. The feasibility of using PD to treat patients with AKI in LRS is now well documented with excellent outcomes being reported in recent publications from Tanzania [14], Sudan [15], and Nigeria [16]. The Saving Young Lives Program has been actively helping support and develop PD programs to treat patients with AKI in LRS [17].

## Conclusion

In summary, the Bagasha et al. study in this issue of *BMC Nephrology* is an important initial step in better characterizing AKI in LRS, using well established, standard definitions and measures. But, we also need good data on community-acquired and non-sepsis related AKI. Additional well-designed, high quality studies of AKI epidemiology will help inform the design of AKI prevention and treatment programs in LRS. These studies will also be important to build political and social support for these programs, as they highlight the high morbidity and mortality associated with AKI in a young, otherwise healthy population.
